# The cycle of neglect: the mpox emergency in the Americas is far from ending

**DOI:** 10.1016/j.lana.2023.100429

**Published:** 2023-01-20

**Authors:** The Lancet Regional Health – Americas

Since May 2022, mpox disease (formerly monkeypox) has been reported across all six WHO regions, accounting for 84330 laboratory-confirmed cases (as of January 06, 2023). Despite the high number of cases initially reported in Europe (the first region outside Africa to report mpox cases), the outbreak was quickly contained using effective contact tracing, treatment, and targeted preventive measures that kept serious outcomes to a minimum. The same did not happen in the Americas. Out of the 74 mpox deaths reported worldwide, 54 happened in the Americas, and the region has the highest disease burden. The Americas house six of the ten most affected countries globally (USA, Brazil, Colombia, Peru, Mexico, and Canada), from which more than 57,000 confirmed cases were reported as of January, 2023. In this special issue of *The Lancet Regional Health – Americas*, we present a collection of papers that explore the clinical, psychological, political, and humanitarian aspects of the current emergency in the region, with a special focus on Brazil and Mexico, and call for unified efforts that aim at a global elimination of mpox, leaving no region behind.

The epidemiology of the ongoing outbreak suggests most transmissions happened through sexual contact, establishing mpox as an emerging sexually transmissible infection (STI). Although surveillance data remain incomplete, most confirmed cases outside Africa have been among men who have sex with men (MSM) and the available information suggests an important intersection between mpox, HIV, and other STIs. The global pattern was reproduced in two case series from Brazil and Mexico, the first detailed reports from Latin American countries. In Brazil, Silva and colleagues found that 89·7% of confirmed cases in Rio de Janeiro were reported by MSM, 53·2% were HIV-positive, 21·2% had syphilis, and 33·0% had other STIs. In a Mexican cohort, Núñez and colleagues found that 97·6% of confirmed cases were reported by MSM, and 52·9% were HIV-positive. The overlap between mpox and other STIs is a silver lining as it can provide impetus for the implementation and strengthening of combined, targeted actions to increase diagnosis and treatment for several co-occurring STIs in high-risk groups. Sadly, the current outbreak has been linked to high levels of stigmatisation of the disease, discouraging suspected cases from seeking diagnosis and treatment, hindering public efforts to eliminate transmission, and fomenting misinformation and discrimination. Similar issues were faced in the early 1980s during the HIV epidemic, and it is unacceptable for us to repeat the same mistakes.

Efforts to contain and eliminate mpox in the Americas are underway, but with varying degrees of success. Some decline in reported cases among MSM in the USA appears to be linked to effective communication and engagement with key communities, paired with vaccination of at-risk populations and case-contacts. However, similar to the early days of the COVID-19 pandemic, the lack of disaggregated data may hide the disproportionate impact and unequal access for certain groups. Nsoesie and Vu cite the case of Georgia, USA, where in August, 2022, Black residents made up 79% of the positive mpox cases but only 45% of the recipients of the mpox vaccine Jynneos. In Brazil, the lack of leadership commitment to the emergency may have favoured the explosion of cases. Homophobic declarations by then-President Bolsonaro during the worst moments of the outbreak cemented the stigmatisation of the disease, and the country now has a shortage of the only nationally approved treatment (Tecovirimat) and delayed arrival of vaccine doses. In this special issue, Scheffer and colleagues and Gadelha and colleagues discuss the case of the mpox response in Brazil and propose actions that could help the region overcome the health emergency. Reduced access to emergency-approved antivirals and the smallpox vaccine (MVA-BN) is a recurrent issue for all Latin American and Caribbean (LAC) countries that rely on externally provided drugs and vaccines and undermine public campaigns to contain the outbreak and eliminate transmission. The PAHO Revolving Fund has secured some of the third generation vaccines through Bavarian Nordic to be made available for LAC countries and called for more collaborative action to stop the outbreak in the region.

The multicountry mpox outbreak was declared a Public Health Emergency of International Concern (PHEIC) on July 23, 2022, and the WHO director-general, Dr Tedros Adhanom Ghebreyesus, hopes to be able to declare the end of PHEIC for mpox in 2023. This is based on the current global trend that weekly cases have declined more than 90% from the peak in August, 2022; however, the global picture is not an homogeneous one. If we look at the Americas alone, weekly cases continue to increase in several countries, and the region is the only one still considered at high risk under WHO assessments. Although the global trend indicates that the situation seems to be moving towards resolution, eliminating mpox must be an international effort. Achieving zero new cases in Europe does not solve the problem if American and African countries remain a reservoir of viruses. Neglecting the endemic circulation of mpox in Africa for the past two decades is probably the main reason we are now battling a multicountry health threat. Looking at the emergency through each region's lenses is important for an equitable and population-centred response. With this special issue, we call for a unified response that aims for global mpox elimination to prevent history from repeating itself. No one is safe until everyone is.

